# Bergenin Activates SIRT1 as a Novel Therapeutic Agent for Osteogenesis of Bone Mesenchymal Stem Cells

**DOI:** 10.3389/fphar.2019.00618

**Published:** 2019-06-14

**Authors:** Weiduo Hou, Chenyi Ye, Mo Chen, Weixu Li, Xiang Gao, Rongxin He, Qiang Zheng, Wei Zhang

**Affiliations:** ^1^Department of Orthopedics, Second Affiliated Hospital, School of Medicine, Zhejiang University, Hangzhou, China; ^2^Research institute of Orthopaedics, Zhejiang University, Hangzhou, China; ^3^Department of Rheumatology, Second Affiliated Hospital, School of Medicine, Zhejiang University, Hangzhou, China

**Keywords:** Bergenin, bone mesenchymal stem cells, osteogenesis, SIRT1, bone regeneration

## Abstract

Bone mesenchymal stem cells (BMSCs) are important candidates for bone regeneration. The role of Bergenin, a C-glucoside of 4-O-methyl gallic acid obtained from the species, Bergenia, in BMSC osteogenesis has not yet been elucidated. We therefore investigated the effects of Bergenin on the osteogenesis of BMSCs and found that Bergenin enhanced osteoblast-specific markers and downregulated the adipocyte-specific markers *in vitro*. Furthermore, using a rat calvarial defect model, we found that Bergenin significantly improved bone healing, as determined by imaging and histological analyses. Moreover, it also upregulated SIRT1 expression. A SIRT1 inhibitor (EX 527) decreased the enhanced bone mineral formation caused by Bergenin. Taken together, these findings show that Bergenin accelerated the osteogenic differentiation of BMSCs, at least partly through the activation of SIRT1.

## Introduction

In clinical practice, approximately 5–10% of fractures result in delayed healing or nonunions, followed by morbidities and functional limitations (Dozza et al., 2018). Bone-marrow-derived mesenchymal stem cells (BMSCs) have the potential to differentiate into bone tissue, making them attractive candidates for bone regeneration (Walmsley et al., 2016). Accordingly, it is essential to identify therapeutic strategies to enhance BMSC osteogenesis.

Bergenin is a colorless, crystallin isocoumarin primarily obtained from the species, Bergenia. It is a C-glucoside of 4-O-methyl gallic acid (Barai et al., 2018) and has been reported to engage in antioxidant, anti-inflammatory, antiarthritic, immunomodulatory, antinarcotic, wound-healing, antidiabetic, and *in vitro* neuroprotective activities. Barai et al. (2018) reported that Bergenin might prevent neurodegenerative disorders. Bergenin inhibited methylglyoxal-induced oxidative stress and inflammation-induced cytokine expression in MC3T3-E1 cells (Lee and Choi, 2018). Wang et al. (2017) reported that Bergenin ameliorated experimental colitis in mice by enhancing expression of SIRT1 to inhibit NF-κB-mediated macrophage activation. Based on the promising beneficial role of SIRT 1 on osteogenesis and bone metabolism (Feng et al., 2016;Deng et al., 2017; Zainabadi et al., 2017; Qu et al., 2018;Zhang et al., 2018; Wang et al., 2019), it is of great interest to explore the possible impact of Bergenin on osteogenesis.

To date, the pharmacological actions of Bergenin during osteogenesis have not yet been elucidated. We hypothesized that Bergenin may promote the osteogenic differentiation of BMSCs through the activation of SIRT1. The results of our study showed that Bergenin enhanced the osteogenic differentiation of BMSCs both *in vitro* and *in vivo*.

## Materials and Methods

### Cells and Reagents

Human BMSCs, as previously reported (Zhang et al., 2015), were purchased from Cyagen Biosciences (from multiple healthy adult donators aged 18–45 years, HUXMA-01001, Guangzhou, China). These cells can differentiate into osteoblasts, adipoblasts, and chondrocytes under specific induction conditions. Adherent cells were trypsinized and passaged after reaching 80% confluence (2–3 days after seeding). Cells from passages 3–5 were used in subsequent experiments. As previously reported (Wang et al., 2017), Bergenin (purity > 99%) was purchased from JingZhu Biological Technology (Nanjing, China). EX 527, a SIRT1 inhibitor, was prepared by Selleck Chemicals (Houston, TX, USA). This study was carried out in accordance with the principles of the Basel Declaration and recommendations of Zhejiang University. The protocol was approved by the Animal Ethics Committee of Zhejiang University.

### Cytotoxicity Assay

To evaluate the impact of Bergenin on the viability of BMSCs, CCK-8 (Dojindo, Kumanoto, Japan) assay and 3-(4, 5-dimethylthiazolyl-2)-2,5-diphenyltetrazolium bromide (MTT) assay (Beyotime, Shanghai, China) were applied. The cells (5000/well) were seeded into 96-well plates and allowed to adhere for 24 h. After that, cells were treated with different concentrates of Bergenin. After the treatment for 1, 5, and 7 days, the medium was removed, and the cells were treated with 10% CCK-8 solution or 0.5 mg/ml MTT solution in 100 μl of low-glucose Dulbecco’s Modified Eagle’s Medium without fetal bovine serum for 3 h at 37°C. Absorbance at 450 nm, which was directly proportional to cell proliferation, was measured by a microplate reader (ELX808; BioTek, Winooski, VT, USA).

### Osteogenic Differentiation and Adipogenic Differentiation Protocol of BMSCs

BMSCs (3 × 10^4^/cm^2^) were cultured in complete growth medium (HUXMA-90011, Cyagen Biosciences) and incubated at 37°C under 5% CO_2_. For osteogenic differentiation, the cells were subsequently cultured in osteogenic induction medium (HUXMA-90021; Cyagen Biosciences). For adipogenic differentiation of BMSCs, cells were induced in adipogenic induction medium (HUXMA-90031; Cyagen Biosciences). The cells were maintained by the addition of fresh osteogenic induction medium every 2–3 days.

### Measurement of Alkaline Phosphatase (ALP) Activity

According to a previous study (Zhou et al., 2015a), we first lysed the cells with RIPA lysis buffer (Beyotime, Shanghai, China) to measure the ALP activity of cells. Then, ALP activity was measured using a *p*-nitrophenyl phosphate colorimetric determination (Sigma-Aldrich, Shanghai, China). All tests were performed according to the manufacturer’s protocols. The ALP activity was also measured in blood samples taken at the time of animal sacrifice.

### Alizarin Red (ARS) and Oil Red O Staining

After the induction of osteogenic differentiation, mineral deposition was assessed by ARS (Cyagen Biosciences). Cells were fixed in 4% paraformaldehyde (Sangon Biotech, Shanghai, China) for 15 min at room temperature and then washed with distilled water twice. A 1% solution of Alizarin Red was added and incubated for 10 min at room temperature; this was followed by rinsing with distilled water. Photographs of images were then taken using an inverted microscope (Leica, Wetzlar, Germany).

After the induction of adipogenic differentiation, fat droplet was assessed by Oil Red O staining kit (Cyagen Biosciences). Cells were fixed in 4% paraformaldehyde (Sangon Biotech, Shanghai, China) for 15 min at room temperature and then washed with distilled water twice. An Oil Red O staining was added and incubated for 30 min at room temperature; this was followed by rinsing with distilled water. Photographs of images were then taken using an inverted microscope (Leica, Wetzlar, Germany).

### Immunofluorescence

Cells (3 × 10^4^/cm^2^) were cultured in induction medium in a 12-well plate, and runt-related transcription factor 2 (RUNX2) and SIRT1 were detected using a fluorescence microscope (EU5888; Leica) after 3 days’ induction. Briefly, the cells were fixed in 4% paraformaldehyde for 15 min at room temperature after treatment. They were then blocked for 30 min in 0.01% Triton X-100 and 5% bovine serum albumin. Fixed cells were washed and incubated overnight with anti-RUNX2 (#12556S; 1:400; Cell Signaling Technology, Shanghai, China) and SIRT1 (#8469S; 1:100; Cell Signaling Technology). Cells were then incubated with a fluorescence-conjugated secondary antibody (ab150077 or ab150075, Abcam, Shanghai, China) for 120 min at room temperature, and nuclei were stained with 4’,6-diamidino-2-phenylindole (DAPI; Sigma-Aldrich, Shanghai, China) for 5 min; they were then observed using an inverted fluorescence microscope (Leica).

### Quantitative Real-Time Polymerase Chain Reaction

Total cellular RNA was isolated using RNAiso reagent (Takara Bio, Kusatsu, Japan) and reverse-transcribed into cDNA in a reaction volume of 20 μl with Prime Script RT Master Mix (Takara Bio) according to our previous study (Zhang et al., 2018). After that, 1 µl of cDNA was used as the template for the quantitative real-time polymerase chain reaction (qPCR). All gene transcripts were quantified by PCR using the Power SYBR^®^ Green PCR Master Mix (Takara Bio) on the ABI StepOnePlus System (Applied Biosystems, Warrington, UK). According to the manufacturer’s instructions, the cycle conditions of PCR were as follows: 95°C for 30 s, 40 cycles of 95°C for 5 s, and 60°C for 30 s. The relative target gene expression levels were calculated using the 2−∆∆Ct method. The mRNA of the target genes and the housekeeping gene (GAPDH) were quantified in separate tubes. All primers were synthesized by Sangon Biotech. Primers used are followed: GAPDH, Forward: CGGACCAATACGACCAAATCCG; Reverse: AGCCACATCGCTCAGACACC; ALP, Forward: TTGACCTCCTCGGAAGACACTCTG; Reverse: CGCCTGGTAGTTGTTGTGAGCATAG;RUNX2, Forward: ACTTCCTGTGCTCGGTGCT; Reverse: GACGGTTATGGTCAAGGTGAA; COL1A, Forward: GAGAGCATGACCGATGGATT; Reverse: CCTTCTTGAGGTTGCCAGTC; PPARγ, Forward: GGGATCAGCTCCGTGGATCT; Reverse: TGCACTTTGGTACTCTTGAAGTT; SIRT1 Forward: TAGCCTTGTCAGATAAGGAAGGA; Reverse: ACAGCTTCACAGTCAACTTTGT.

### Western Blot Analysis

Cells were lysed in RIPA lysis buffer (Beyotime) with a proteasome inhibitor (Beyotime). Total proteins were separated by 10% sodium dodecyl sulfate-polyacrylamide gel electrophoresis and then transferred to a polyvinylidene fluoride membrane (Millipore, Shanghai, China). After blocking in 5% nonfat milk for 2 h, the membranes were incubated overnight at 4°C with antibodies specific for glyceraldehyde 3-phosphate dehydrogenase (A00227-1; 1:8,000; Boster Biological Technology, Wuhan, China), RUNX2 (#12556S; 1:1,000; Cell Signaling Technology), PPARγ (#2435S; 1:1,000; Cell Signaling Technology), or SIRT1(#8469S; 1:1,000; Cell Signaling Technology). Horseradish-peroxidase-conjugated goat anti-rabbit IgG (BA1056; 1:5000; Boster Biological Technology) was used as a secondary antibody for 2 h at room temperature. The immunoreactive bands were detected using an enhanced chemiluminescent detection reagent (Millipore, Shanghai, China). Signal intensity was measured using a Bio-Rad XRS chemiluminescence detection system (Bio-Rad, Hercules, CA, USA).

### *In Vivo* Evaluation of Rats

The accelerated bone-forming ability of Bergenin was assessed in a calvarial defect model in Sprague–Dawley rats (Aghaloo et al., 2007; Sun et al., 2014). All experiments were conducted in accordance with the Animal Care and Use Committee guidelines of Zhejiang province and the Institutional Animal Care and Use Committee of Zhejiang University. Three-month-old male (approximately 200 g) Sprague–Dawley rats were obtained from the Academy of Medical Sciences of Zhejiang province. According to our previous studies (Ye et al., 2018; Zhang et al., 2018), rats were anesthetized with 0.3% sodium pentobarbital (Sigma-Aldrich) intraperitoneally at 30 mg/kg body weight. A trephine drill was utilized under constant irrigation to create a 4-mm, critically sized defect in the parietal bone. Care was taken to avoid injury to the underlying dura mater. All rats received the above surgical procedures. The rats were divided randomly into two groups: a control group (sham) and an experimental (Bergenin) group (*n* = 6/group). To conduct effective statistical analysis, sample size ≥ 4/group is required. In this study, *n* = 6/group was set, which was consistent with our previous study (Chen et al., 2017) and other published studies (Chen et al., 2017; Deng et al., 2018; Wang et al., 2018). As reported in previous studies (Gao et al., 2015; Yun et al., 2015; Wang et al., 2017), the Bergenin group was intraperitoneally treated with Bergenin in phosphate-buffered saline (PBS) at 50 mg/kg body weight weekly after surgery, throughout the 8 weeks; the sham group was treated with an equal volume of PBS.

The rats were sacrificed in a CO_2 _chamber at 8 weeks after surgery. The cranium was collected for radiographic and histological analyses, and the serum was assessed for ALP activity.

### Microcomputed Tomography Evaluation

To evaluate callus formation and bridging bone formation at bone defect sites 8 weeks postoperatively, the craniums were scanned using a μCT-100 imaging system (Scanco Medical, Brüttisellen, Switzerland) with X-ray energy settings of 70 kVp, 1,024 reconstruction matrix, 14.8-μm slice thickness, and an exposure time of 300 ms. According to previous studies (Shinozaki et al., 2014; Toda et al., 2014), after three-dimensional (3D) reconstruction using the manufacturer’s software was conducted, a square region of interest (ROI) centered on the area of the defects was selected for further qualitative and quantitative analyzes. The bone volume fraction (bone volume/total volume, BV/TV) was calculated by 3D standard microstructural analysis.

### Histological Evaluation

Samples were fixed with 4% paraformaldehyde for 24–48 h at room temperature and decalcified using 10% EDTA (Sigma-Aldrich) with a solution change once weekly for more than 8 weeks at 4°C before embedding in paraffin. Serial sections with a thickness of 5 μm were cut and mounted onto polylysine-coated slides. Consistent with previous studies (Shinozaki et al., 2014; Toda et al., 2014), the cross section of the central area of the defects was serially cut at 5 μm thick for further histological evaluation. Hematoxylin and eosin and Masson staining were performed separately on consecutive tissue sections, as described in our previous study (Zhang et al., 2016).

### Statistical Analysis

Statistical analysis was performed using SPSS statistical software for Windows, version 19.0 (IBM, Armonk, NY, USA). All experiments were performed in at least triplicate, and the data are presented as the mean ± standard deviation. Statistical significance was determined using a two-tailed Student’s *t* test when comparing two groups and by a one-way analysis of variance followed by Bonferroni’s *post hoc* test when comparing more than two groups. A value of *P* ≤ 0.05 was considered to represent a statistically significant difference.

## Results

### Bergenin had no Adverse Effect on the Viability of BMSCs

**Figure 1:**
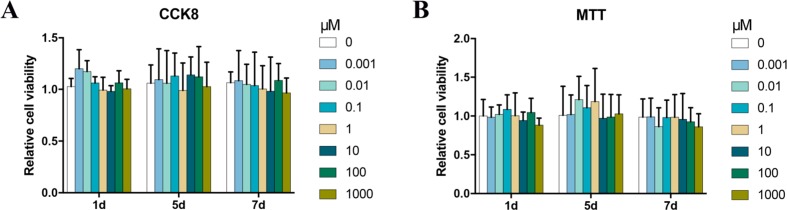
Effects of Bergenin on cell activity in human bone marrow mesenchymal stem cells (BMSCs). **(A)** The effects of Bergenin on BMSC viability were detected using Cell Counting Kit-8. **(B)** The effects of Bergenin on BMSC viability were detected using MTT assay. Data are expressed as the mean ± standard deviation (SD), *n* = 3. *P < 0.05 vs. BMSCs without Bergenin.

To determine the cytotoxic potential of Bergenin, its effects on BMSC viability were evaluated by the CCK-8 and MTT assay. No significant cytotoxic effect was observed between groups treated with and without Bergenin (**Figure 1**).

### Bergenin Upregulated the Levels of Osteo-Specific Markers Under Osteogenic Conditions

**Figure 2:**
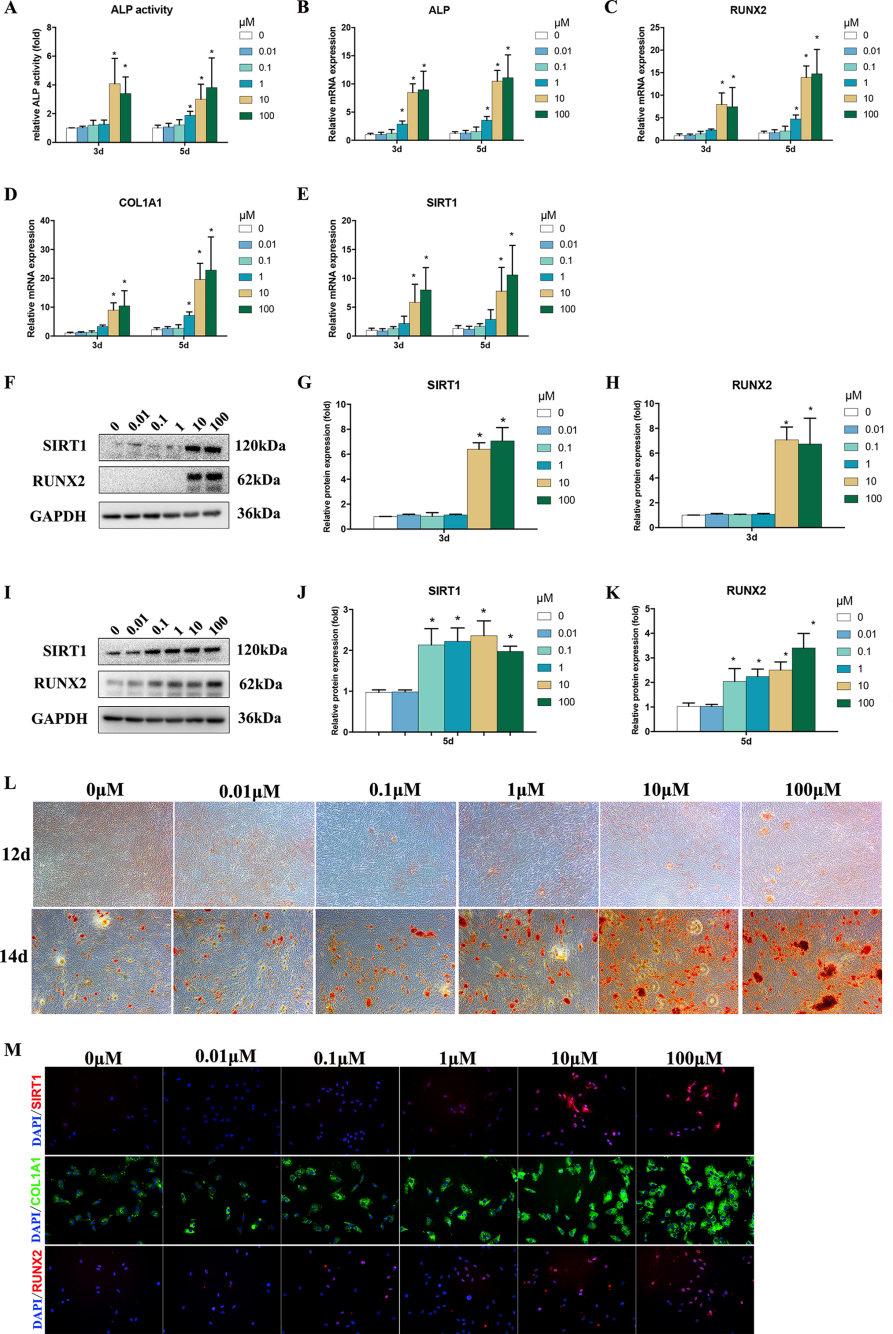
Effects of Bergenin on osteogenesis of bone marrow mesenchymal stem cells (BMSCs). **(A)** The effects of Bergenin on alkaline phosphatase activity at days 3 and 5 during the osteogenic differentiation of BMSCs. **(B**–**E)** mRNA expression of RUNX2, ALP, and COL1A1 was determined by quantitative reverse transcription polymerase chain reaction at day 3 and day 5 during osteogenic differentiation. mRNA expression levels were normalized to GAPDH. **(F**–**K)** The expression of RUNX2 and SIRT1 protein was determined by Western blot analysis after osteogenic differentiation at days 3 and 5. Protein expression levels were normalized to glyceraldehyde-3-phosphate dehydrogenase. Data are expressed as the mean ± standard deviation (SD) of three independent experiments, and one of three independent experiments is shown. Data are expressed as the mean ± SD, *n* = 3. *P < 0.05 vs. BMSCs treated with osteogenic induction medium alone. **(L)** Alizarin red staining at days 12 and 14 of osteogenic differentiation. Magnification ×40. **(M)** Immunofluorescence staining showing that the protein levels of RUNX2, COL1A1, and SIRT1 are upregulated by the addition of Bergenin (10 or 100 μM) at day 3 of osteogenic differentiation. COL1A1 is stained green. RUNX2 and SIRT1 are stained red. Nuclei are stained with 4′,6-diamidino-2-phenylindole (blue). Magnification ×200.

ALP activity is an important marker for the osteogenesis of BMSCs. After treatments using 1–100 μM Bergenin, the ALP activity of the experimental group was increased on days 3 and 5 after the induction of osteogenic differentiation, compared with that of the control group (**Figure 2A**).

To assess the role of Bergenin in the osteogenic differentiation of BMSCs, the levels of osteo-specific genes and proteins, including ALP, RUNX2, and COL1A1, were determined. The qPCR analysis revealed that the ALP, RUNX2, and COL1A1 mRNA levels were significantly increased on days 3 and 5 after the induction of osteogenic differentiation in BMSCs in the presence versus the absence of Bergenin (1 or 100 μM) (*P* < 0.05; **Figure 2B–E**).

Western blot analysis revealed that RUNX2 protein expression was increased by 10–100 μM Bergenin treatment on day 3 after the induction of osteogenic differentiation. Moreover, on day 5, there was a higher level of RUNX2 protein expression in BMSCs treated with certain doses of Bergenin, when compared with those from the control group (**Figure 2F–K**). Moreover, Alizarin Red staining showed significantly more calcium deposits in the 1–100 µM Bergenin treatment group (**Figure 2L**). Using immunofluorescence analysis, we also found higher levels of Runx2 and COL1A1 due to Bergenin on day 3 (10 or 100 µM) (**Figure 2M**).

### Bergenin Downregulated the Levels of Adipo-Specific Markers Under Adipogenic Conditions

**Figure 3:**
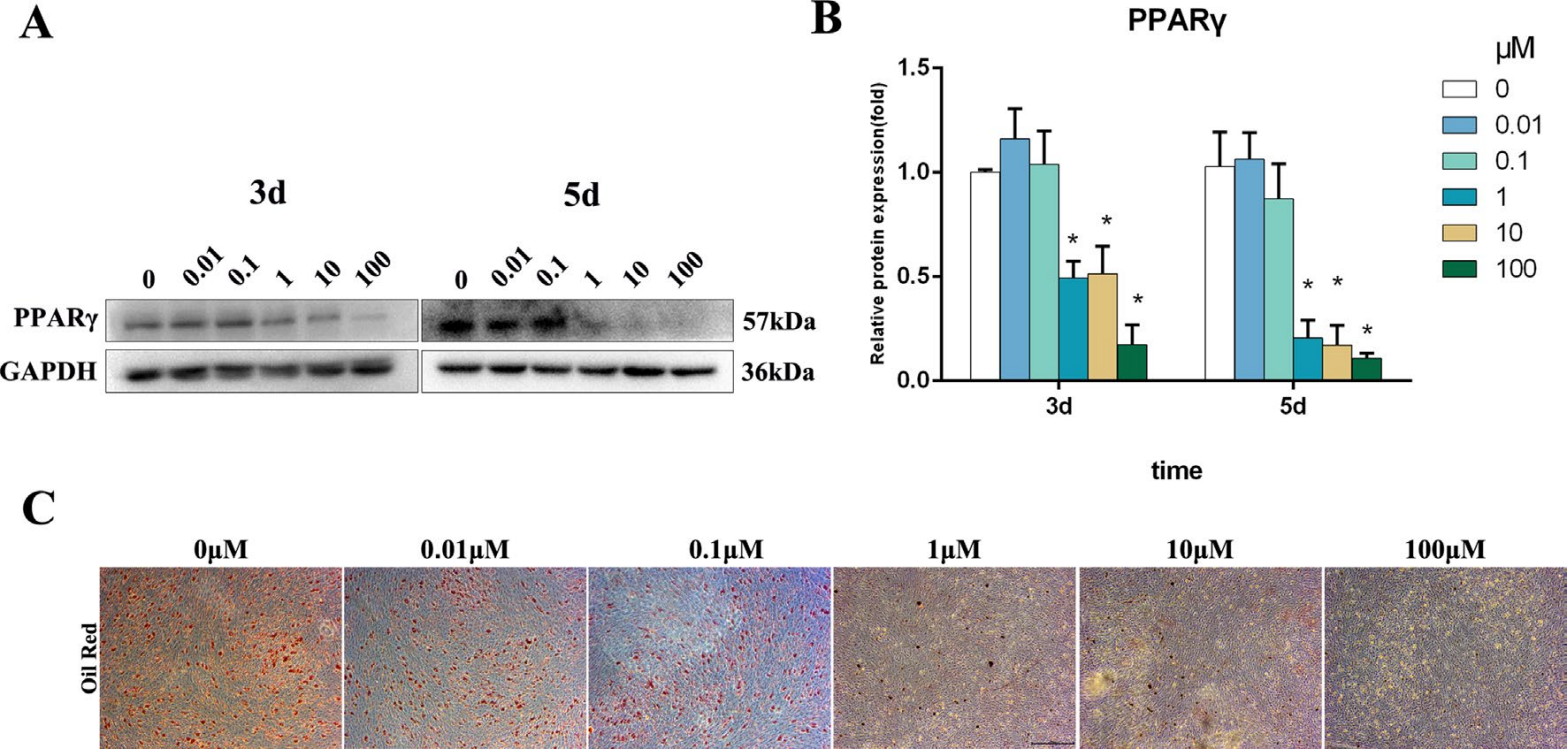
Effects of Bergenin on adipogenesis of bone marrow mesenchymal stem cells (BMSCs). **(A** and **B)** The expression of PPARγ protein was determined by Western blot analysis after adipogenic differentiation at days 3 and 5. Protein expression levels were normalized to glyceraldehyde-3-phosphate dehydrogenase. *P < 0.05 vs. BMSCs treated with osteogenic induction medium alone. **(C)** Oil Red O staining at days 14 of adipogenic differentiation. Magnification ×40.

PPARγ is a master regulator of adipogenic differentiation of BMSCs. Western blot analysis revealed that PPARγ protein expression was decreased by 1–100 μM Bergenin treatment on days 3 and 5 after the induction of osteogenic differentiation (**Figure 3A and B**). Furthermore, oil Red O staining found significantly less fat droplet in the 1–100 µM Bergenin treatment group (**Figure 3C**).

### Bergenin Activated SIRT1 Expression

Based on the vital beneficial role of SIRT1 on osteogenesis (Feng et al., 2016; Deng et al., 2017; Zainabadi et al., 2017; Qu et al., 2018; Zhang et al., 2018; Wang et al., 2019) and the results of previous studies (Wang et al., 2017), we measured the SIRT1 mRNA and protein levels. The results of qPCR and Western blot analyses indicated lower expression of SIRT1 in the control group, compared with certain dose of the Bergenin-treated group (**Figure 2D and E**). Moreover, using immunofluorescence analysis, we also found increased levels of SIRT1 due to Bergenin on day 3 after the induction of osteogenic differentiation (10 or 100 µM) (**Figure 2H**).

### Accelerated Osteogenic Differentiation of BMSCs Due to the Presence of Bergenin was Partially Impaired by a SIRT1-Specific Inhibitor (EX 527)

**Figure 4:**
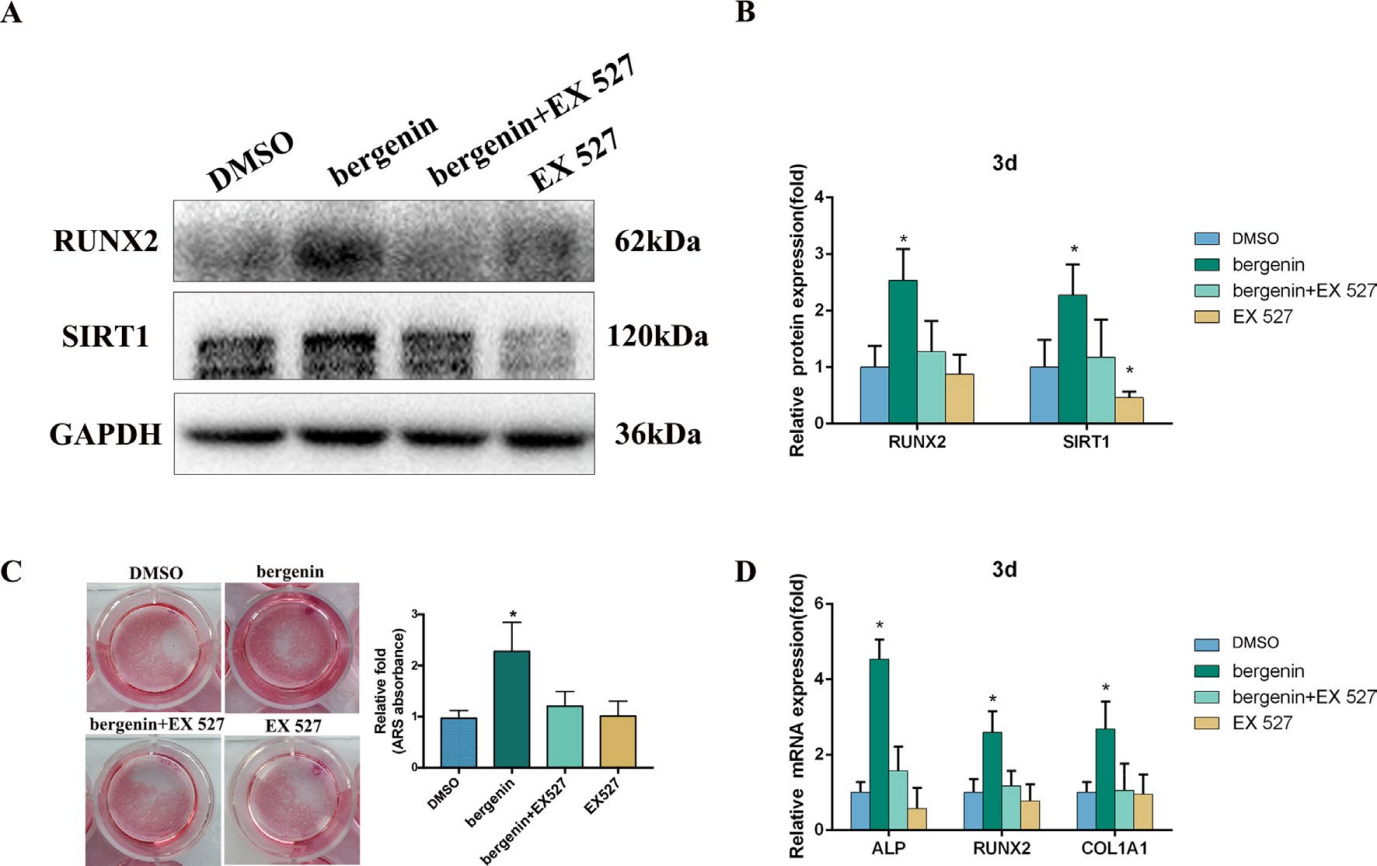
Effects of a SIRT1-specific inhibitor (EX 527) on the enhanced expression of an osteoblast-specific gene of bone marrow mesenchymal stem cells by Bergenin. **(A** and **B)** The expression of RUNX2 and SIRT1 in blank, control + EX 527, Bergenin (10 μM), and Bergenin (10 μM) + EX 527 groups was determined by Western blot analysis. EX 527 (10 μM) was applied for 1 h, followed by culture in osteogenic induction medium with Bergenin for 3 days. Protein expression levels were normalized to glyceraldehyde-3-phosphate dehydrogenase. Data are expressed as the mean ± standard deviation (SD) of three independent experiments, and one of three independent experiments is shown. Data are expressed as the mean ± SD. *P < 0.05 vs. group with osteogenic induction medium alone. **(C)** Alizarin red staining and quantification of mineralization at day 12 of osteogenic differentiation. **(D)** The mRNA expression of RUNX2, ALP, and COL1A1 in blank, control + EX 527, Bergenin (10 μM), and Bergenin (10 μM) + EX 527 groups was determined by quantitative reverse transcription polymerase chain reaction. EX 527 (10 μM) was applied for 1 h, followed by culture in osteogenic induction medium with Bergenin for 3 days. mRNA expression levels were normalized to GAPDH. *P < 0.05 vs. BMSCs treated with osteogenic induction medium alone.

To confirm the role of SIRT1, we investigated the effect of its specific inhibitor (EX 527) on the osteogenic differentiation of BMSCs. According to previous studies, EX-527 is the most specific and potent SIRT1 inhibitor (Li et al., 2011; Sasca et al., 2014; Nikseresht et al., 2018). EX-527 (10 μM) was added to osteogenic induction medium (Li et al., 2011); after 3 days, the expression of osteo-specific markers was determined. **Figure 4A and B** shows that lower levels of RUNX2 were found in the Bergenin + inhibitor-treated cells compared with cells treated with Bergenin alone. Blocking SIRT1 also decreased the levels of mineralization (**Figure 4C**). In a similar manner, the downregulation of SIRT1 by EX-527 inhibited the expression of osteo-specific genes (*ALP*, *RUNX2*, and *COL1A1*) (**Figure 4D**).

### Bergenin Accelerated Bone Formation in a Calvarial Defect Model in Rats

**Figure 5:**
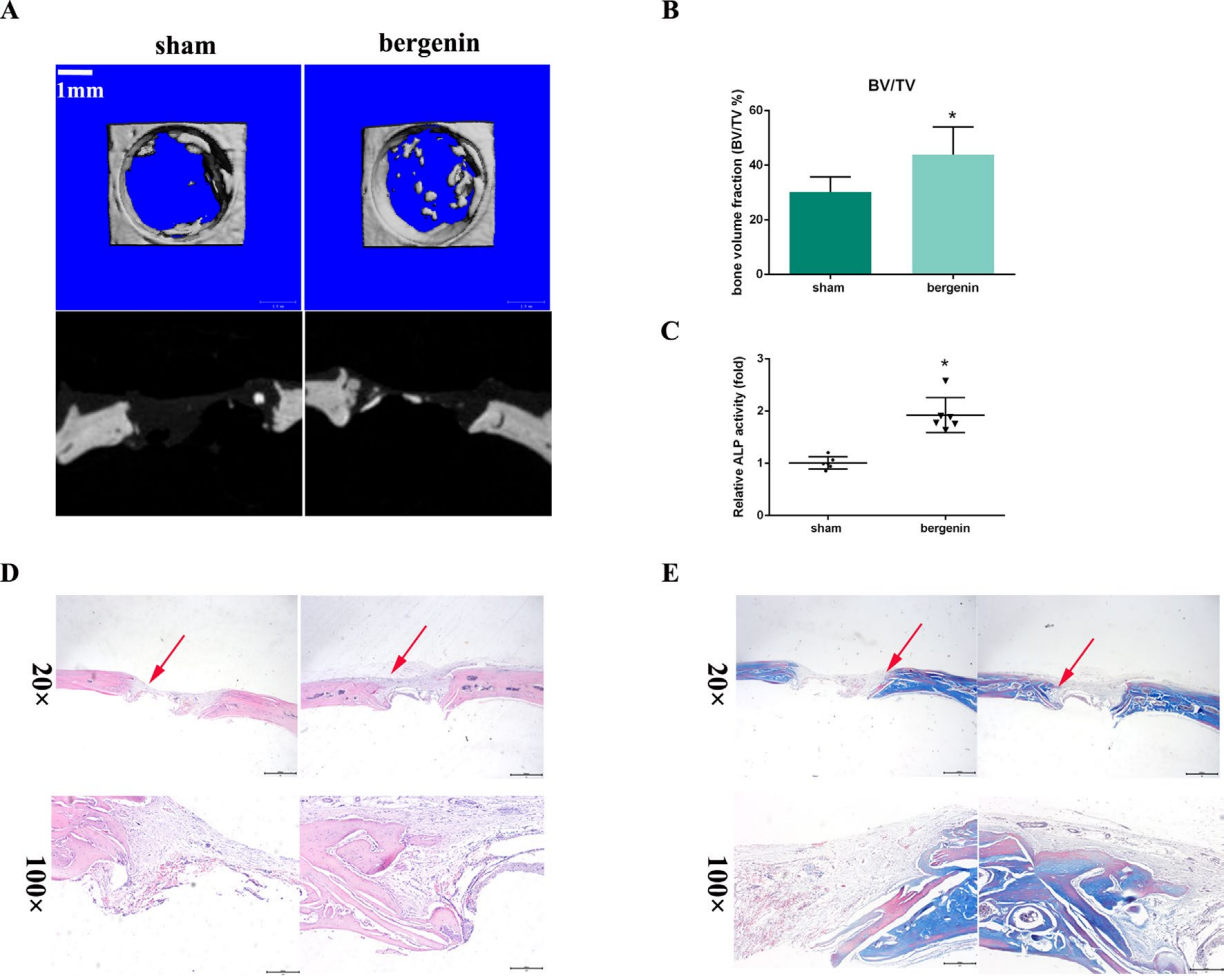
Bergenin accelerated bone formation in a calvarial defect model in rats. **(A)** Microcomputed tomography analysis for bone healing. **(B)** Bone volume was analyzed by microcomputed tomography. Data are expressed as the mean ± standard deviation (SD). Reactions were performed in triplicate. *P < 0.05 vs. bone defects with PBS (sham group). **(C)** ALP activity in serum, *P < 0.05 vs. bone defects with PBS (sham group). **(D)** Histologic analysis for bone healing. HE, hematoxylin and eosin staining; Magnification ×40 (bar = 500 μm) and ×100 (bar = 200 μm). **(E)** Masson, Masson’s trichrome staining. Magnification ×40 (bar = 500 μm) and ×100 (bar = 200 μm).

To assess the *in vivo* effect of Bergenin on osteogenesis, a rat calvarial defect model was used. The morphology of new bone formation was characterized using microcomputed tomography (micro-CT) analysis. Representative images are shown in **Figure 5A**. Micro-CT revealed that the Bergenin group showed increased bone formation in the calvarial defect compared with the sham group at 8 weeks after surgery. Quantiﬁcation of the mineralized areas also showed a signiﬁcant increase in mineralization tissues in the Bergenin group (**Figure 5B**). Additionally, higher levels of ALP activity were detected in the serum of the Bergenin group (**Figure 5C**).

Representative histological photographs of each group, including hematoxylin and eosin and Masson’s trichrome staining, are shown in **Figure 5D and E**. In the Bergenin group, a thick callus consisting of newly formed woven bone tissue was observed in the defect area, with a narrower distance between bone defects, compared with the control animals.

## Discussion

To the best of our knowledge, this is the first demonstration that the SIRT1 agonist Bergenin effectively promoted both the *in vitro* and *in vivo* osteogenesis. First, it promoted the osteo-specific markers and mineralization forming of BMSCs and inhibited the adipogenesis *in vitro*. It also enhanced bone formation in a bone defect model and upregulated the expression of SIRT1. Moreover, blocking the activation of SIRT1 decreased the enhanced osteogenesis of BMSCs due to the presence of Bergenin. Taken together, these findings indicated that Bergenin accelerated the osteogenic differentiation of BMSCs, at least partly through upregulation of SIRT1.

Bone defects and nonunions occur frequently in clinical settings. Bergenin is one of the plant-derived chemical constituents in traditional medicine and engages in multiple biological activities. Importantly, previous studies have reported that it can affect bone metabolism. Nazir et al. (2007) reported that it showed antiarthritic activity through the possible modulation of the Th1/Th2 cytokine balance. Kwang et al. revealed that Bergenin had protective effects on methylglyoxal-induced cytotoxicity in MC3T3-E1 osteoblasts (Suh et al., 2018). Pretreatment with Bergenin before methylglyoxal exposure reduced mitochondrial dysfunction, indicating that Bergenin may prevent the development of diabetic osteopathy (Lee and Choi, 2018). In our study, we found that Bergenin upregulated the levels of osteo-specific markers (ALP, RUNX2, and COL1A1) and accelerated the mineralization of BMSCs *in vitro*. Moreover, increased bone formation was found after Bergenin treatment *in vivo*. Of note, numerous studies have demonstrated that fat-induction factors inhibit osteogenesis, and, conversely, bone-induction factors hinder adipogenesis (Chen et al., 2016a; Chen et al., 2016b). Likewise, in our study, we found that Bergenin could inhibit the levels of adipo-specific marker (PPARγ) and suppress the lipid droplet formation.

Increasing evidence has demonstrated that SIRT1 can promote bone formation (Cohen-Kfir et al., 2011; Chen et al., 2014; Qu et al., 2016). Tseng et al. (2011) reported that resveratrol promoted the osteogenesis of human mesenchymal stem cells by upregulating RUNX2 gene expression *via* the SIRT1/FOXO3A axis. Moreover, SIRT1 also inhibits adipogenesis (Zhou et al., 2015b). A previous study indicated that Bergenin could improve the expression of SIRT1 by inhibiting inflammation (Wang et al., 2017). Consistent with these results, we also found that Bergenin acted as an activator of SIRT1. Bergenin upregulated protein and mRNA expression of SIRT1 in a dose-independent manner. Furthermore, the accelerated osteogenic differentiation of BMSCs due to the presence of Bergenin was partially impaired by the addition of a SIRT1-specific inhibitor (EX 527).

Our study has several limitations. First, although our results indicated that Bergenin upregulated SIRT1 to enhance BMSC osteogenesis, the underlying mechanism remains unclear. Second, as the long-term dose–response relationship and safety of Bergenin were not adequately characterized, the translational relevance of these findings needs to be confirmed. In addition, in this study, we did not compare the *in vivo* results with the positive control group, which absolutely causes osteogenesis, by classic drugs or gold standard clinically. Future studies are required.

## Conclusion

Based on our data, we found that Bergenin enhanced the osteogenic differentiation of BMSCs, partly through activation of SIRT1. Bergenin may therefore be a novel therapeutic agent for the treatment of bone defects.

## Ethics Statement

All experiments were conducted in accordance with the Animal Care and Use Committee guidelines of Zhejiang province and the Institutional Animal Care and Use Committee of Zhejiang University.

## Author Contributions

WZ and QZ contributed to the design and funding sources to this study. WZ, WH, and MC drafted the manuscript. WL, XG, RH, and CY did all the *in vitro* and *in vivo* parts of the study. All authors have contributed significantly and read and approved the final manuscript.

## Funding

This study was supported by a grant from the National Natural Science Foundation of China (No. 81802221, No. 81672147, No. 81501906, and No. 81572124), China postdoctoral science foundation (2018M640567) and the Zhejiang Provincial postdoctoral preferred foundation (zj20180131).

## Conflicts of Interest Statement

The authors declare that the research was conducted in the absence of any commercial or financial relationships that could be construed as a potential conflict of interest.
